# Exosomes as Powerful Engines in Cancer: Isolation, Characterization and Detection Techniques

**DOI:** 10.3390/bios11120518

**Published:** 2021-12-16

**Authors:** Marwa Gamal Saad, Haluk Beyenal, Wen-Ji Dong

**Affiliations:** The Gene and Linda Voiland School of Chemical Engineering and Bioengineering, Washington State University, Pullman, WA 99164, USA; marwa.aly@wsu.edu (M.G.S.); beyenal@wsu.edu (H.B.)

**Keywords:** exosomes, isolation, characterization, detection, biomarkers, communication, cancer

## Abstract

Exosomes, powerful extracellular nanovesicles released from almost all types of living cells, are considered the communication engines (messengers) that control and reprogram physiological pathways inside target cells within a community or between different communities. The cell-like structure of these extracellular vesicles provides a protective environment for their proteins and DNA/RNA cargos, which serve as biomarkers for many malicious diseases, including infectious diseases and cancers. Cancer-derived exosomes control cancer metastasis, prognosis, and development. In addition to the unique structure of exosomes, their nanometer size and tendency of interacting with cells makes them a viable novel drug delivery solution. In recent years, numerous research efforts have been made to quantify and characterize disease-derived exosomes for diagnosis, monitoring, and therapeutic purposes. This review aims to (1) relate exosome biomarkers to their origins, (2) focus on current isolation and detection methods, (3) discuss and evaluate the proposed technologies deriving from exosome research for cancer treatment, and (4) form a conclusion about the prospects of the current exosome research.

## 1. Introduction

There are two types of extracellular vesicles released from cells: exosomes and ectosomes. Exosomes have smaller diameter sizes than ectosomes [1] and are released via the fusion of multivesicular bodies with the plasma membrane, whereas ectosomes are shed directly from the plasma membrane [2]. Exosomes are endosome-derived membrane vesicles with a size range of 20–150 nm [3,4,5,6,7,8,9,10,11,12] and are derived naturally from nearly all cell types [1,13,14,15]. When exosomes were first discovered in 1981 in mammalian cells, they were believed to act as discharged vesicles of obsolete molecules [16,17]. After their roles in communication were revealed, they were thought to communicate only within one species, but they were later found to engage in interkingdom communication [10]. Due to their roles in cellular communications, exosomes are carriers for vital biomarkers that originate from parental cells. These biomarkers include nucleic acids, e.g., miR-21, which is considered a reference biomarker for ovarian [18], prostate [19], and breast cancers [20]. As a result, exosomes are considered unique biomarkers for the diagnosis and prognosis of various malicious diseases, such as cancer.

Cancer is one of the deadliest diseases worldwide. Prostate cancer ranks as the most widespread cancer, and it causes a significant percentage of death [21]. Lung cancer, the second-most deadly cancer, causes 25% of cancer deaths with either small cell carcinoma or non-small-cell carcinoma [22]. Cortical cancer is the second-most fatal cancer and the third-most widespread one [23], and breast cancer is a hostile tumor among women over 40 [24,25]. Due to the high fatality of cancers and complicated treatment procedures, it is highly desired to find a reliable cancer biomarker to improve cancer detection for early diagnosis and treatment [24,25,26]. Since cancer-derived exosomes are involved in both the development and metastasis of cancer through intracellular communication [27,28], are stable with small size phenotypes, and are accessible in most biological fluids [29,30], they are considered excellent candidates for early cancer diagnosis and vehicles for cell-based therapy and drug delivery [10].

This review will start with a brief description of exosome structure, release, and biogenesis mechanisms, followed by a description of the known techniques for isolation and detection. The exosome applications for cancer will be specified. The multiple roles of exosomes, such as (1) biomarkers, (2) diagnostic agents, and (3) signal transduction factors, will be included. Finally, future research efforts to address the technical challenges related to exosome study will be discussed.

## 2. Exosome Structure, Release, and Biogenesis

### 2.1. Exosome Structure

**Figure 1:**
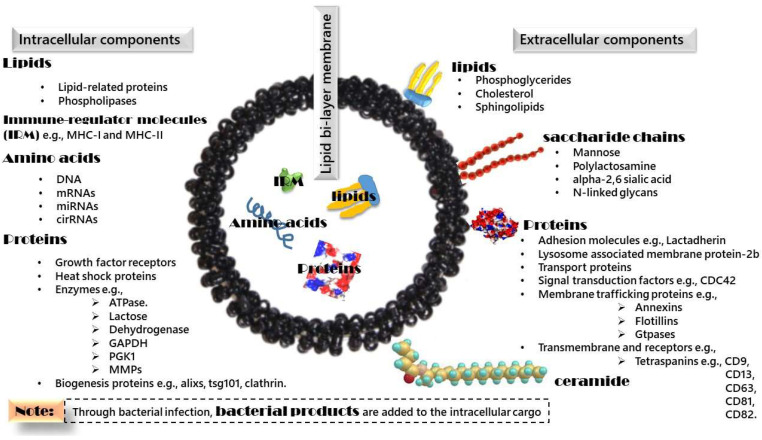
The exosome structure in mammalian cells.

In this section, we will emphasize the structure of exosomes in mammalian populations. Exosomes derived from mammalian populations contain a lipid bilayer membrane [31], intracellular components, and extracellular components bound to the outer membrane (Figure 1).

The lipid bilayer membrane consists of two layers of phospholipids, each of which contains lipids in a head and tail form. The head is hydrophilic, and the tail is hydrophobic. With this polarity characteristic, the lipid bilayer is a continuous barrier surrounding the intracellular components.

The intracellular components include (1) amino acids, e.g., DNA, messenger RNA (mRNA), microRNA (miRNA), and circular RNA (cirRNA); (2) proteins, e.g., growth factor receptors, heat shock proteins, enzymes, and biogenesis proteins; and (3) lipids, e.g., lipid-related proteins and phospholipases [32,33,34,35,36,37,38]. 

The extracellular components include (1) lipids, e.g., phosphoglycerides with long and saturated fatty-acyl chains, cholesterol, and sphingolipids (to provide structure rigidity) [39,40]; (2) ceramide a differentiated component between exosomes and lysosomes [41,42]; (3) saccharide chains, e.g., mannose, polylactosamine, alpha-2,6 sialic acid, and N-linked glycans; and (4) proteins, e.g., lactadherin and membrane transport and fusion proteins such as annexins, flotillins, GTPases, and tetraspanins. 

To conclude, mammalian cells secrete exosomes with a lipid bilayer membrane to protect the intracellular components, which are considered the messages that exosomes carry and transfer between parent and recipient cells. The extracellular components are the signals used for specific cell receptors to facilitate cellular communication between exosomes and receiving cells. 

### 2.2. Exosome Biogenesis

**Figure 2:**
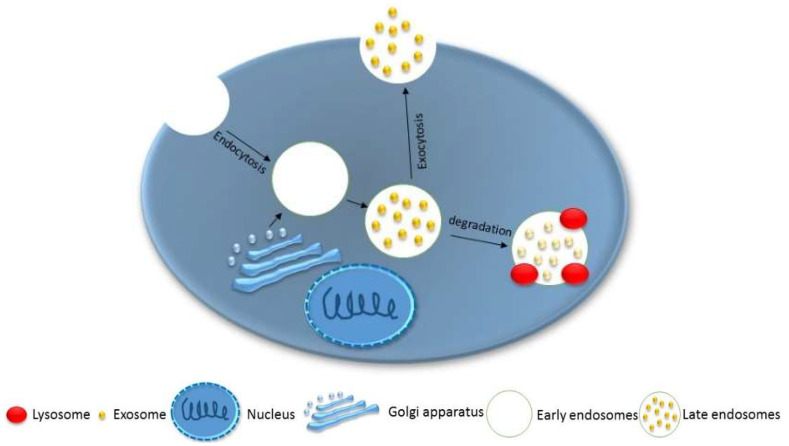
Exosome biogenesis in mammalian cells.

Exosome biogenesis was first discovered in 1983, when Harding, Heuser, and Stahl imaged the process in rat reticulocytes using gold transferrin temperature-dependent label methodology [43]. As transferrin uptake is a temperature-dependent process [44], their method started at 4 °C to enhance its binding with the plasma membrane, then increased the temperature to 37 °C to facilitate intracellular vesicle production. These intracellular vesicles were found to be labeled after engulfing the gold transferrin. Finally, these multivesicular bodies fused with the plasma membrane and released the gold transferrin outside the cells by exocytosis. These studies [43,44] suggested that exosome biogenesis in mammalian cells starts when cells engulf intracellular fluids and that tube-shaped early endosomes are formed and develop to have intraluminal vesicles by engulfing cytosolic components such as transmembrane and peripheral proteins; then, spherical mature endosomes are located close to the nucleus. Afterwards, these mature endosomes (also called multivesicular bodies (MVBs)) fuse with the plasma membrane to secrete exosomes into the outer membrane environment (Figure 2) [4,45]. Most mammalian cells, e.g., dendritic cells, epithelial cells, mesenchymal stem cells, and cancer cells, secrete exosomes in healthy and unhealthy conditions. In addition, body fluids, e.g., serum, saliva, urine, and breast milk, contain exosomes [46,47,48].

### 2.3. Exosome Release

The exosome release process starts when the MVBs fuse with the plasma membrane. Several mechanisms were proposed to understand the exosome release process. Under normal conditions, the endosomal sorting complex required for transport (ESCRT) machinery is critical for exosome release [49]. The ESCRT machinery consists of four protein complexes, numbered as 0, I, II, and III, associated with the AAA ATPase Vps4 complex [50]. It was found that the depletion and/or knocking down of certain proteins in the ESCRT machinery affected the rate of exosome release. For example, the exosome secretion increased because of knocking down ESCRT-III, CHMP4C, VPS4B, VTA1, and ALIX, whereas the secretion decreased when ESCRT-0 proteins, e.g., Hrs and TSG101, and ESCRT-I protein, e.g., STAM1, were depleted [51].

In addition, ESCRT-independent mechanisms were proposed for exosome release in the case of ESCRT machinery being knocked down [52]. Tetraspanin proteins, e.g., CD9, CD63, and CD82, enhanced the exosome secretion of β-catenin from HEK293 cells [53,54,55]. In addition, targeting specific lipid enzymes such as neutral sphingomyelinase 2 to modify the plasma membrane lipid configuration (size of the headgroup, length, and saturation of the acyl chains) inhibited exosome secretion [41]. Exosome release is also regulated and stimulated by multiple factors, such as Ca^2+^ [56], ceramide synthesis [41], and acidosis [57].

The p53-based mechanism was proposed to operate under stress conditions. It was found that the production of exosomes under stress conditions was regulated by the p53 protein to communicate to other cells to respond to stress in a phenomenon called the “bystander effect” [58,59]. TSAP6 is upregulated and transcribed in response to stress [60]. TSAP6 is a p53-regulated gene. Then, p53 induces cells to secrete specific proteins within exosomes to migrate to other cells, communicate, and face the stress [61]. Yu and colleagues [62] examined the protein secretion in exosomes after a p53-mediated stress response to lung cancer cells in culture. They tested cells containing a wild-type p53 gene (H460) and mutated cells (having a mutant p53 allele). The cells were irradiated with gamma irradiation to induce p53 and apoptosis. They observed a dramatic increase in exosome production as a response to the p53-regulated mechanism due to irradiation. Exosomes were not detected in the cases of the mutant p53 allele or nonirradiated cells. 

The other mechanisms involved a variety of stress stimuli. For example, researchers at the School of Medicine at Flinders University showed the enhancement effect of hypoxia on a percentage of cancer-derived exosomes. In their study, after hypoxia exposure, the exosomes were isolated and quantified using a nanoparticle tracking analysis and immunoblotting for the CD63 and miRNA-210 assays by RT-PCR. They demonstrated that hypoxia enhanced the release of breast cancer-derived exosomes [63]. Additionally, Németh’s research team investigated the effect of the antibiotic ciprofloxacin on exosome release and showed that a low concentration of ciprofloxacin caused the release of DNA proteins on exosome surfaces and blocked them from further cellular processes [64]. Finally, Rab proteins, e.g., Rab 11, Rab 27a,b, and Rab 35, were found to play key regulatory roles in exosomes released in mammalian cells [56,65,66].

These mechanistic studies strongly suggest that exosome release is a stimuli-based process. Further research studies are needed for verification. Once these mechanisms are verified, it will be a starting point for maximizing the production of the desired exosomes and improving the exosome applications.

## 3. General Techniques for Exosome Isolation, Characterization, and Detection

**Figure 3:**
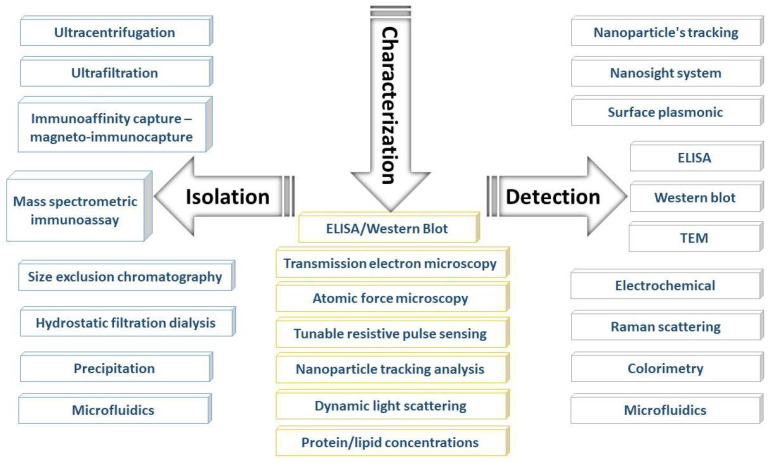
Isolation and characterization techniques for exosomes.

Exosome isolation and detection are a challenge because of the low concentrations and cell line-dependent heterogeneity of the exosomes [67,68,69]. Accordingly, developing and improving reliable methods to prepare, detect, and analyze exosomes is critical for exosome research and will have a great impact on the development of exosome-based disease diagnoses and therapeutics. Figure 3 summarizes the general methods for exosome isolation and detection.

### 3.1. Isolation Techniques

The isolation of exosomes from a cell culture depends mainly on the physical and chemical properties of the exosomes. Ultracentrifugation and ultrafiltration target the size and density of exosomes, and chemical precipitation and immune affinity target specific extracellular proteins. To choose the suitable isolation technique, the number and volume of the samples, the available instruments, and the aim of the analysis must be considered [70]. Here, we will discuss the isolation methods and the advantages and disadvantages associated with each method (Table 1). The isolation methods are classified according to (1) specificity, as specific or nonspecific, and (2) sample volume, as high-throughput or low-throughput. All methods of isolation are considered nonspecific, except for the immune affinity-based techniques. The immune affinity-based techniques and microfluidics are considered low-throughput methods because of their small sample volumes.

#### 3.1.1. Ultracentrifugation

**Figure 4:**
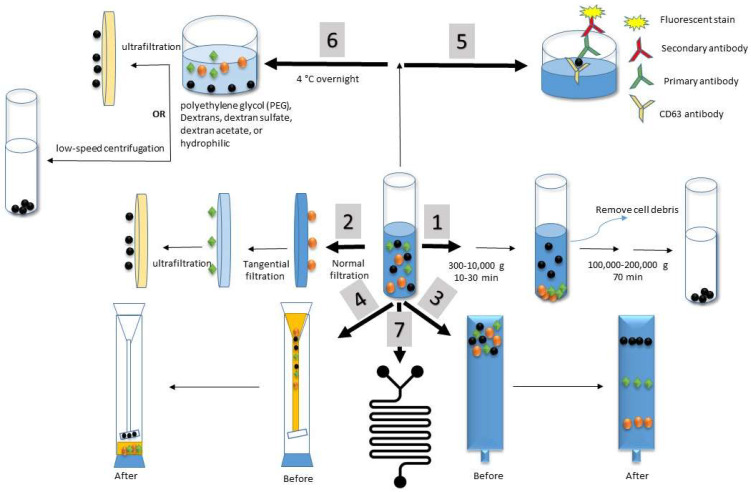
Isolation techniques for exosomes. The exosomes are represented by the small, black balls. The techniques are represented as separated pathways as follows: (1) ultracentrifugation, (2) ultrafiltration, (3) size exclusion chromatography, (4) hydrostatic filtration dialysis, (5) immunoaffinity, (6) precipitation, and (7) microfluidics.

Ultracentrifugation is considered the gold standard for exosome isolation [8,31]. It depends on the size of the particles and the viscosity of the solution. It is always done as a series of centrifugation steps, starting with low-speed centrifugation to remove the cell debris, then high-speed centrifugation up to 100,000× *g* for the precipitation of exosomes [31], as illustrated in Figure 4 pathway no. 1. The isolation efficiency of this technique depends on the g force, the rotor rotation, the angle of the sedimentation force, and the sample viscosity [76]. While this method is simple and easy to follow, with no need for pretreatments, it is time-consuming, and ultrapowerful centrifugation could affect the exosome structure and function [63,76,77]. Exosomes isolated with ultracentrifugation are highly pure with low yields (5–40%) [8,70,78].

#### 3.1.2. Ultrafiltration

The isolation of exosomes by ultrafiltration is based on the exosome size. Membrane filters are used in a series of steps: first, normal filtration to eliminate large components; second, tangential filtration using a molecular weight cutoff membrane to separate proteins from all other contaminants; and lastly, ultrafiltration using a 100-nm track-etched filter to isolate exosomes [74,79], as illustrated in Figure 4 pathway no. 2. The advantages of this method are that (1) it is fast, (2) it does not require special equipment, (3) it is scalable, and (4) it produces a high yield. The challenges associated with this method are the deformation and breaking up of large vesicles due to the force and errors resulting from the unavailability of the exosomes if they attach themselves to the membrane [80]. 

Ultracentrifugation can be combined with filtration with a commercially available nanomembrane concentrator with a uniform size of 13 mm at 3000× *g*. The orientation of the membrane within the receptacle minimizes the shearing force. The weakness of this method is the difficulty of recovering the proteins from the membrane [81].

#### 3.1.3. Size Exclusion Chromatography

Size exclusion chromatography (SEC) isolates exosomes based on particle size in a porous stationary phase. Thus, particles with small hydrodynamic radii will pass through the pores, and exosomes with large hydrodynamic radii will not, as illustrated in Figure 4 pathway no. 3. Early research was done by Baranyai et al. to isolate exosomes from rates and human plasma samples using SEC [82]. Plasma samples were diluted and loaded onto the system. The authors tested various column matrices, e.g., Sepharose 2B, Sepharose CL-4B, and Sephacryl S-400. Their results indicated that the Sepharose CL-4B and Sephacryl S-400 columns were sufficient for significantly reducing the albumin contamination. Their protocol helps to isolate highly purified exosomes with preserved biological activities. Research efforts such as combining SEC with ultracentrifugation to enrich the yield [83] and combining it with ultrafiltration to enhance its efficacy and speed [84] have been done to overcome SEC challenges such as slowness, the need for dedicated equipment, low yield, and difficulty in scaling up [72]. 

#### 3.1.4. Hydrostatic Filtration Dialysis 

Exosomes have been isolated according to their size using a hydrostatic filtration dialysis (HFD) system forced with a low hydrostatic pressure. In 2014, Musante et al. efficiently isolated diabetic nephropathy biomarker-based exosomes from urine samples using a dialysis system [85]. Their dialysis system consisted of a defined 1000-kDa cutoff dialysis membrane connected to a funnel with a long, sheer column that created a hydrostatic pressure to push the solution through the dialysis membrane, as illustrated in Figure 4 pathway no. 4. The system was refilled with pure water until all the pigments were washed out from the dialysis part. This method was found to be simple, fast, and effective: It reduced the labor, maintained the protein pattern, and was capable of processing large sample volumes: 10 mL–1 L with a rate of 75 mL/h.

#### 3.1.5. Immunoaffinity

Antigen–antibody linkage is the main mechanism for the immunoaffinity method, in which specific antigens are used to target specific extracellular proteins on exosome membranes. Technically, the immunoaffinity method can be considered an upgrade of the main enzyme-linked immunosorbent assay (ELISA) mechanism, in which two antibodies are used to detect a specific antigen, as illustrated in Figure 4 pathway no. 5. The first antibody is the antigen-trapping molecule, and the second antibody is the fluorescence-detecting molecule. To enhance the proficiency of this method, two techniques were proposed: the microplate-based immunocapture technique and the immunoaffinity capture/magneto-immunocapture technique. These techniques are further illustrated in the next subsections.

#### Microplate-Based Immunocapture Technique

Briefly, in the microplate-based immunocapture technique, the exosomes are attached directly to a microplate surface. The surface of this microplate is immobilized with the required antibodies to capture exosomes, leading to the exosomes precipitating from the culture [86]. However, the samples must be prepared before treatment, and it is required that there be at least 20 µg of protein content in the exosomes. This technique is highly specific and yields a high RNA content from a low sample volume, as little as 100 μL of sample, compared to the 2.5 mL needed for ultracentrifugation [87]. A novel dendrimer–PEG antibody dual-layer platform was proposed to significantly capture and isolate tumor exosomes from serum samples. This platform was assembled as a sandwich with two layers of carboxylated generation 7 poly amidoamine dendrimers and was stuffed with polyethylene glycol (PEG) (2, 5, and 20 kDa) conjugated with dendrimers. The dendrimers for the bottom layer coated an epoxide-functionalized glass slide. This structure facilitated the multivalent capture ability by applying multiple antibodies and minimizing the nonspecific bindings. This platform possesses high avidity, specificity, antibody orientation flexibility, and tumor-derived exosome yield [88].

#### Immunoaffinity Capture/Magneto-Immunocapture

To add value to the microplate-based technique, magnetic beads, such as latex beads and nano-sized beads, have been conjugated with antibodies [5,35]. One example is Dynabeads. Dynabeads^®^ are superparamagnetic polystyrene beads with a diameter of 1–4.5 µm. These beads are specified to conjugates with the anti-human CD63 antibody, either directly or via a secondary linker such as anti-mouse IgG [8]. Using this new combination of antibody and magnetic particles increases the capture affinity and sensitivity and makes it easy and rapid to proceed. The efficiency of this method depends on the interaction between the antigen and antibody, temperature, concentration of exosomes, and incubation time [5]. Sample volumes could be scaled up or down without any restriction. The isolation yield is 15 times higher than with ultracentrifugation [12]. Although this method is considered the superior strategy for isolating exosomes from cell culture media, it depends on the quality of the pre-enriched exosomes [5]. 

An ideal example is T-cell Immunoglobulin Mucin Protein (Tim4) binding with phosphatidylserine molecules on the surfaces of exosomes. Tim4 immobilized on magnetic beads has Ca^2+^-dependent binding to phosphatidylserine. Moreover, exosomes can be released from the Tim4 surface by adding a complexing agent to remove Ca^2+^ [89]. Greening and coworkers (2015) [12] evaluated the efficacy of three isolation techniques: ultracentrifugation (UC-Exos), OptiPrep™ density gradient centrifugation (DG-Exos), and immune isolation using EpCAM (CD326) antibodies coupled to magnetic beads (IAC-Exos) targeting markers Alix, TSG101, and HSP70 to enrich exosomes released from LIM1863 human colon cancer cells. The isolated exosomes had a uniform size of 40–150 nm, and they verified that the IAC method was the most efficient for exosome isolation.

#### 3.1.6. Precipitation

Based on the chemical properties of exosomes: (1) a water-excluding polymer, e.g., polyethylene glycol (PEG); (2) dextran derivatives, e.g., dextran sulfate and dextran acetate; and (3) hydrophilic polymers such as polyvinyl alcohol, polyvinyl acetate, and polyvinyl sulfate were used to chemically precipitate exosomes from the culture [8]. After a mixed sample was incubated at 4 °C overnight with the precipitation solution, exosomes could be isolated from the precipitate either by low-speed centrifugation or filtration, as illustrated in Figure 4 pathway no. 6. This method is easy-to-handle, does not require specific equipment, and can be scalable for large sample volumes. However, if the samples are not precleaned of cells and cellular debris, proteins and polymeric materials will be found as coprecipitates [8].

A modified protocol was proposed by Alvarez et al. 2012 [70]. The authors used ExoQuick-TC to precipitate exosomes [63]. Their protocol is perfect for proceeding with multiple samples in the absence of an ultracentrifuge and for targeting RNAs and mRNAs for biomarker identification [70].

#### 3.1.7. Microfluidics

Multiple microfluidic chips have been designed to isolate exosomes rapidly and efficiently with significant reductions in the sample volume, reagent consumption, and isolation time, as illustrated in Figure 4 pathway no. 7. However, scalability, validation, sample pretreatments, and standardization are considered disadvantages for these devices [90]. 

Wang and colleagues (2017) [9] fabricated an acoustofluidics device to isolate exosomes directly from undiluted blood samples based on their size and density using ultrasound standing waves. With respect to the channel orientation, particles are subjected to acoustic force and pushed toward the pressure node. The device consists of two modules. The first separates larger components, >1 μm in diameter, such as red and white blood cells, and platelets with 99% efficiency. The second module isolates exosomes to 98.4% purity. This device offers continuous flow exosome isolation while maintaining the structures, characteristics, and functions of the exosomes. Additionally, it enables short processing times with decreasing human intervention.

In another device, exosomes with diameters of 40–100 nm were preferentially trapped on a ciliated micropillar with a porous silicon nanowire. Proteins and other cellular debris were filtered out. Exosomes were released from the porous silicon nanowires by dissolving them in a phosphate buffer solution. ExoChip is a commercial immune-microfluidics chip that is functionalized with a commonly expressed antigen, CD63 (a member of the tetraspanin family). The specific interaction between the CD63 and antibodies immobilized on the chip allowed the isolation of exosomes from mixed cultures. While, in the integrated microfluidic exosome device, the sample was mixed with antibody-labeled magnetic beads; then, a lysis buffer was added, and detection reagents were introduced in a separate chamber. To improve the scalability of the integrated device, in-line ultraviolet and dynamic light scattering detectors were coupled with the field–flow fraction system to isolate and characterize exosomes rapidly [91].

Dr. Chang’s research group fabricated microfluidics chips to isolate exosomes from plasma and cell culture samples based on an ionic exchange property. In 2017, they fabricated an integrated platform to isolate exosomes using an ion-selective membrane [92]. One year later, they upgraded their system by adding a pressure-driven flow force to filter out unwanted debris before concentrating exosomes on the ion-selective membrane [93]. This system was fast and sensitive and recovered 60–80% of the exosomes from the serum and cell culture compared to 25% for other systems.

To summarize this section, multiple techniques have been recognized for isolating exosomes from the culture, e.g., ultracentrifugation, ultrafiltration, size exclusion chromatography, hydrostatic filtration dialysis, immunoaffinity, precipitation, and microfluidics. These techniques have been modified and/or combined to improve the isolation procedure. 

### 3.2. Characterization and Detection Techniques

Generally, analyses of the characteristics of purified exosomes fall into four basic categories: size, concentration, purity, and content. For size and purity, transmission electron microscopy (TEM) is the standard, with a very low-throughput method for taking and analyzing data [81]. More recently, the NanoSight system [94] has been used to image and determine particle sizes and concentrations. A promising approach to assessing the purity is combining NanoSight with a protein assay [95], but this is a source-dependent method. Exosome contents can be examined using the “-omics” methods, such as proteomics, transcriptomics (miRNA or mRNA), lipidomics, and glycomics (glycoproteins), or analyzed using more focused methods, such as Western blot, RT-qPCR, and GC-MS [76]. Recently, protein and/or lipid concentration assays using simple spectrophotometer protocols have been considered promising methods for characterizing the protein and lipids contents of exosomes [96].

The method of characterization is chosen according to the purpose of the analysis. If the purpose is to identify the morphology and confirm the sample purity, then TEM is the standard method to follow. If the purpose is to determine the size and morphology of the particles, then a nanoparticle tracking analysis is sufficient. Western blot and ELISA can be used to detect and identify proteins with respect to their role (up- or downregulation). Spectrophotometry is a standard method for determining the concentrations of the particles. Table 2 details these methods with respect to their targets, advantages, and disadvantages.

Recently, several individual approaches and combined methods, such as surface plasmonic biosensors [29,103,104], microchip-based technologies [14,105], electrochemical techniques [106], fluorescence [107], and colorimetry [108], have been proposed for exosome detection; some of them have been upgraded over time to reach the high-throughput, high sensitivity, and real-time detection and quantification of disease-based exosomes. These methods are summarized in Table 3.

Examining all this information on the characterization and detection techniques used for exosome samples indicates that visualizing the procedure with respect to the chemical composition of the particles and preparing the samples are the main factors in choosing the suitable technique. However, it is better to combine two or more techniques to confirm the results.

## 4. Tumor Exosomes for Cancer Detections

Exosomes are found to promote cancer angiogenesis, generate the premetastatic niche, and modulate the host immune system [49]. In the next few sections, we will summarize the cases in which exosomes have been used for diagnosis and for monitoring cancer agents.

### 4.1. Exosomes as Disease Biomarkers with Diagnostic Potential

With respect to cancer, exosomes have potential effects on cancer development and tissue reprogramming [115]. Exosome nucleic acids pool and proteins act as the primary biomarkers for early cancer early detection and diagnosis [1,116,117].

Exosomes have a cell line-based structure that suggests a subpopulation distribution on a cell line basis. The role of these subpopulations is likely related to the normal and cancer cells and helps in diagnostic purposes. Exosomes released from normal and cancer cell lines have different (1) nucleic acid contents and (2) membrane structures in accordance with their cholesterol contents, surface proteins, and cholesterol: phospholipid ratios [67,118].

Exosomes contain functional components, e.g., RNA, DNA, and proteins, which can be used as biomarkers for diagnostic and monitoring purposes and can be easily transferred to recipient cells. Exosomes derived from infected cells mimic special elements spanning the normal cells that provide a blueprint of tumor cells for medical purposes [119].

The miRNA is one of the critical intracellular components in exosomes. It is a class of noncoding RNA with 18–25 nucleotides that plays vital roles in cell-to-cell communication pathways in carcinogenesis [120]. These noncoding RNAs can facilitate metastasis by enhancing the molecular pathways associated with cancer [121]. Systemically, miRNAs are the most abundant species, with around 42.3% of the exosome RNA pool [122]. Other RNA fractions include rRNA, tRNA, noncoding RNA, piwi-interacting RNA, small nuclear RNA, and small nucleolar RNA. The common miRNAs are miR-22-3p, miR-99a-5p, miR-99b-5p, miR-124-3p, and miR-128 [13]. It was suggested that miRNAs play vital roles in physiological processes such as RNA splicing, protein phosphorylation, chromosomal abnormality, and angiogenesis [13,122]. The exosomal miRNA profiles can potentially be used as cancer biomarkers, e.g., miRNA-141, miRNA-200a, and miRNA-200c [18,40,123,124].

Deoxy ribonucleic acid (DNA) is another critical component of exosome structures. ExoDNA is poorly studied compared to ExoRNA. Previously, it was believed that microvesicles have intracellular single-stranded DNA (ssDNA) and mitochondrial DNA [125]. Thakur et al. 2014 found evidence that exosomes have intra- and extracellular double-stranded DNA (ddDNA) as a whole genomic material [126]. They compared the types and concentrations of the DNA loops in pretreated exosome samples with dsDNase and untreated samples. They observed a significant reduction in the concentration of the DNA loop in the treated samples, which means that exosomes carry high concentrations of ddDNA. They also found that the circulating exosomal ddDNA was a promising tumor-based mutation biomarker that could be used to validate cancer diagnostics and prognostics by identifying multiple genes, such as EGFR, BRAF, RAS, IDH, and HER2, because (1) it is stable, (2) it is biocompatible, and (3) its functional group can be modified [127,128]. ExoDNA is a key regulator for tumor immunity [128]. Cancer cells secrete harmful fragmented DNA through their exosomes to avoid senescence (cell death) and avoid the stimulator of interferon genes (STING) and cyclic GMP–AMP synthase (cGAS) resulting from DNA accumulation [129]. STING and cGAS are two machineries that are activated by DNA accumulating in the cytoplasm. These DNA machineries act against tumorigenesis [130]. The therapeutic efficacy of the tumor is based on the STING mechanism [131]. The loops of DNA fragments that have accumulated in the cytoplasm because of radiotherapy and chemotherapy induce the antitumor response and STING activation in dendritic cells to prevent further tumor growth and promote inflammation. The mechanism of packing the DNA inside the exosomes is still unclear. Exosome biogenesis is enhanced in infected cells because of the hypoxia and the low pH [132]. 

The third critical diagnostic biomarker component of the exosomes is their proteins, as they are protected from the proteinases and stable in plasma and serum circulation. There are specific types of exosomal proteins that act to discriminate between different cell types; for example, the epithelial cell adhesion molecule (EpCAM) differentiates between cancer cells and normal cells. Other types of proteins that differentiate exosomes from other vesicles are secreted by all cell types. These biomarkers include CD9; CD63; CD81; LAMP1; heat shock proteins (Hsp25, Hsp60, Hsp70, and Hsp90); synthenins; endosomes; and calnexin [5,12,133,134,135,136]. 

To conclude this section, lists of specific biomarkers according to either the disease or the cell type is given in Table 4 and Table 5 [137,138]. Interestingly, it was found that the same biomarker could be used as a reference for several diseases—for example, miR-21 is used for ovarian cancer [18], prostate cancer [19], and breast cancer [20]—and that exosomes released from different cell lines could include identical biomarkers [139].

### 4.2. Exosome-Based Technologies for Cancer Detection and Identification

Much research has been done on the detection and quantification of exosomes derived from prostate, breast, lung, colorectal, and ovarian cancers. Cancer-derived exosomes can be extracted from plasma, serum, cell lines, and urine. Urine is the most significant source for cancer-derived exosomes, as it is safe, easy to manipulate, and cost-effective [147]. Researchers at the School of Medicine at Cardiff University investigated the possibility of using urinary exosome biomarkers as tools for monitoring therapy. They found that PSA, PSMA, and 5T4 biomarkers could be quantified from exosomes derived from urine prostate cancer [150]. Sampling of the cancer-derived exosomes is a challenge, because they are tiny and are present in low concentrations. In the following section, we will briefly discuss these research efforts based on their techniques.

#### 4.2.1. Surface Plasmonic Biosensor Technology

**Figure 5:**
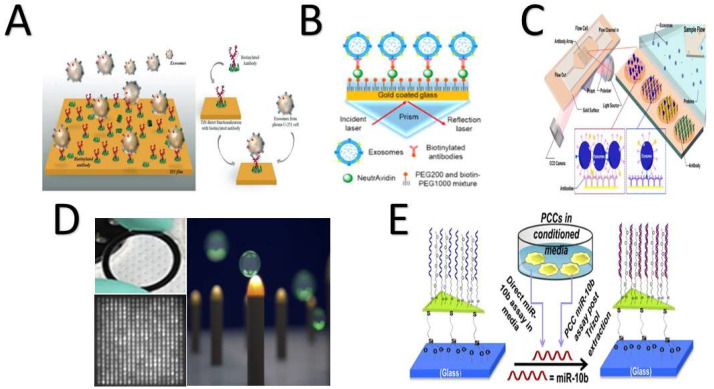
Examples of surface plasmonic biosensors for exosome detection and quantification. The figure permissions are as follows: (**A**) License number 5071041050010, license date 16 May 2021, licensed content publisher John Wiley and Sons. Licensed content publication of “Advanced Functional Materials”, licensed content title “Detection of Glioma-Derived Exosomes with the Biotinylated Antibody-Functionalized Titanium Nitride Plasmonic Biosensor”. (**B**) Adapted with permission from Liu, C. et al. 2018. Sensitive detection of exosomal proteins via a compact surface plasmon resonance biosensor for cancer diagnosis. ACS sensors, 3(8), pp. 1471–1479. Copyright (2021) American Chemical Society, Rightslink^®^ by Copyright Clearance Center. (**C**) Adapted with permission from Zhu, L. et al. 2014. Label-free quantitative detection of tumor-derived exosomes through surface plasmon resonance imaging. Analytical chemistry, 86(17), pp. 8857–8864. Copyright (2021) Label-Free Quantitative Detection of Tumor-Derived Exosomes through Surface Plasmon Resonance Imaging. Analytical Chemistry (acs.org) and further permissions related to the excerpted material should be directed to ACS. (**D**) Adapted from Raghu, D. et al. 2018. Nanoplasmonic pillars engineered for single exosome detection. PloS ONE, 13(8), p. e0202773, with no permission required for reuse, as the work was made available under the Creative Commons CC0 public domain dedication. (**E**) Adapted with permission from Joshi, G.K et al. 2015. Label-free nanoplasmonic-based short noncoding RNA sensing at attomolar concentrations allows for the quantitative and highly specific assay of microRNA-10b in biological fluids and circulating exosomes. ACS nano, 9(11), pp. 11075–11089, hps://pubs.acs.org/doi/abs/10.1021/acsnano.5b04527, 10/2021, and further permissions related to the excerpted material should be directed to ACS.

Surface plasmonic biosensor (SPB) technology uses optical base devices as label-free detectors to monitor and quantify protein interactions. It is considered a promising technique for detecting biomarkers in cancer-derived exosomes. This technology includes a versatile visual toolbox for sorting various tightly packed biological species (Figure 5). Both the electromagnetic field of the surface plasmon and the optical waves originating from the mass oscillations of the electronics are responsible for the charge density of thin (nanoscale) metallic films. This technology offers labor savings, label-free miniaturization, and sensitivity [29,103,104]. Zhu fabricated a biosensor based on detecting exosome surface proteins using SPB technology with antibody microarrays with no need for enrichment or purification methods for cancer diagnosis purposes [116]. Another surface plasmon biosensor chip was developed to monitor exosomes derived from pancreatic cancer by detecting microRNA-10b. It is composed of a synthesized nano-Au prism attached to a single-nucleotide miRNA sensor on the surface of the plasmon resonance biosensor. This is an ultrasensitive device that provides specificity and sensitivity, but exosomes must be highly purified in order to be quantified using this platform [120]. Researchers in Switzerland focused on using multiple antigens to instantly capture and identify exosomes derived from breast cancer from three cell lines and characterize their disease development [27]. They developed an immunosensor with antibody-functionalized surface plasmon resonance biosensing, in which multiple antigens are combined in a gold-coated layer for the kinetics label-free monitoring of molecular interactions. They used their biosensor to screen two exosome biomarkers (CD9 and CD63) and four cancer biomarkers (CD24, CD44, EpCAM, and human epidermal growth factor receptor 2 (HER2)). Several advantages are associated with this methodology, such as clinical applicability, flexibility, usability, and sensitivity for detecting low sample concentrations. 

Liu updated a similar platform to detect exosomes derived from lung cancer and overcome the high cost associated with the fabrication process. His platform is composed of an Au-coated glass layer conjugated with a prism and a NeutrAvidin–polyethylene glycol (PEG)–thiol–biotin PEG mixture. Exosomes are captured on the surface of that mixture, and then, the laser signals are reflected through the prism. This platform offers high sensitivity and simplicity, but the detection of biomarkers differs according to the sample origin for the same disease, e.g., the signals of the exosomal epidermal growth factor receptor (EGFR) were not the same in human serum as in the cell lines [30]. Another surface plasmon biosensor chip was developed with a nonfactionalized nanogold layer to distinguish between exosomes and extracellular macrovesicles associated with lung cancer in mice, which revealed the importance of surface [29] exosome properties [186]. A surface biotinylated antibody-functionalized titanium nitride plasmon resonance biosensor was fabricated to detect glioma-derived exosomes (glioma is a brain cancer that starts in the glial cells of the brain or the spine). The titanium nitride biosensor was able to detect both CD63 and epidermal growth factor receptor variant-III with detection limits of 10^−3^ μg mL^−1^ and 2.75 × 10^−3^ μg mL^−1^, respectively. In addition, it had excellent performance, stability level, and biocompatibility with titanium nitride [174]. Research groups in Australia and Singapore have developed a real-time-functionalized ani-HER2 surface plasmon biosensor to detect breast cancer cells. Their platform is simple, label-free, and sensitive, with a detection limit of 8.2 × 10^−3^ particles/µL [159].

In 2020, Portela created an upgraded nanoplasmonic biosensor featuring nanogap antennas by employing the colloidal lithography process. Gaps that had a size of ~11.6–4.7 nm formed between gold nano-disk pairs. This antenna biosensor detected lung cancer biomarker miRNA-210 via a hybridization assay of DNA/miRNA. Several advantages were reported for this platform, including (1) high performance, (2) high sensitivity, (3) simplicity, (4) cost-effectiveness, (5) a low detection limit (5.1 ng mL^−1^), and (6) the direct detection of miRNAs [176]. Recently, a simple plasmonic surface polydopamine-functionalized Au nanobiosensor with two aptamers was invented. The DNA tetrahedron probes were immobilized on the gold nanoparticle samples under alkaline pH. Afterwards, a covalent bond between aptamer 1 and aptamer 2 was structured as a NH_2_–COOH bond. In the first step, SMMC-7721 exosomes were captured on the surface of the first aptamer, which was complementary to the DNA tetrahedron probes. Then, the second aptamer recognized the SMMC-7721 on the captured exosomes and enhanced the signal amplification. Consequently, signal amplification improved when the first aptamer reduced the HAuCl_4_. This platform offers specificity, a low detection limit of 5.6 × 10^5^ particles/mL, and no need for pretreatments [169].

Exosome–antibody kinetics were studied and described as the hit–stay–run reaction by Yang and coauthors [187]. They created an interferometric plasmonic microscopy with which they were able to image single exosomes, monitor the adsorption of exosomes onto Au surfaces, and determine the exosomal size distribution. This offers the ability to distinguish between exosomes and liposomes [187]. A second real-time detection protocol was designed to detect circulating proteins on exosome surfaces. This mechanism starts with antibodies capturing exosomes on the surface of a plasmonic sensor, which causes a change in the refractive index between the central aperture and nanogroove rings, which changes the intensity of the transmitted light. This technique can detect a sample concentration of 3.86 × 10^8^ exosomes/mL, providing the opportunity to monitor and analyze biomolecular binding kinetics. It can be coupled to a smartphone as a healthcare device [188]. Recently, a smartphone-based sensor was applied to detect single exosomes directly based on their physical and biomolecular structures. This plasmonic biosensor is structured with gold nanoshells at which the same exosomes will be captured and identified by their dimensions and biomolecular structures, such as miRNAs and proteins. This platform is fast, sensitive, and wash-free [189].

#### 4.2.2. Microchip-Based Technology

**Figure 6:**
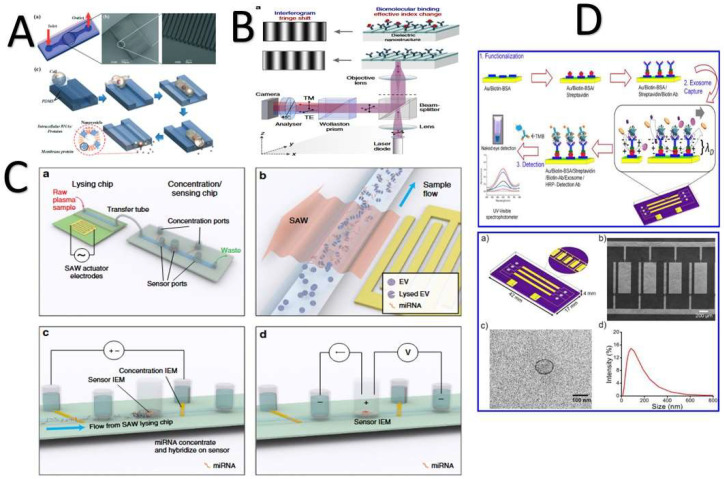
Examples of microfluidic chips for exosome detection and quantification. Figure permissions are as follows: (**A**) License ID 1119355-1, license date May 16, 2021, licensed content publisher the Royal Society of Chemistry. Licensed content publication of “Microfluidic fabrication of cell-derived nanovesicles as endogenous RNA carriers”. (**B**) Adapted from Barth, I., Conteduca, D., Reardon, C., Johnson, S., and Krauss, T.F. 2020. Common path interferometric label-free protein sensing with resonant dielectric nanostructures. Light: Science & Applications, 9(1), pp. 1–9, with no permission required for reuse as the work was made available under Creative Commons CC0 public domain dedication, 10/2021 http://creativecommons.org/licenses/by/4.0/. (**C**) Adapted from Ramshani, Z. et al. 2019. Extracellular vesicle microRNA quantification from plasma using an integrated microfluidic device. Communications biology, 2(1), pp.1–9, with no permission required for reuse as the work was made available under Creative Commons CC0 public domain dedication, 10/2021 http://creativecommons.org/licenses/by/4.0/. (**D**) Adapted with permission from Vaidyanathan, R. at al. 2014. Detecting exosomes specifically: a multiplexed device based on alternating current electrohydrodynamic induced nanoshearing. Analytical chemistry, 86(22), pp. 11125–11132. Copyright (2021) American Chemical Society. Rightslink^®^ by Copyright Clearance Center.

Microchip-based technology is used for circulating, capturing, and detecting exosomes, because it is (1) easy to use, (2) reagent-saving, and (3) highly efficacious [105]. Microchip-based technology has all these advantages, but it also has operating challenges, such as a low-mass transfer scale and interference with exosomal binding [143] (Figure 6). Starting in the USA in 2014, a group of researchers fabricated a multiple-channel chip based on sample transmission through antibody-functionalized arrays with periodic holes and as imaging setup for simultaneous density detection. By applying the fabricated microfluidics chip, they were able to detect CD24 and EpCAM in 20 ovarian cancer patient samples compared to 10 noncancer samples [144]. Their chip provides (1) quantitative analyses of specific exosomes, (2) high sensitivity, (3) retrievability for further analysis, and (4) a one-step process. A research group at the Australian Institute for Bioengineering and Nanotechnology fabricated a multichannel device for multiplexed simultaneous naked eye readouts and UV spectrophotometer quantifications of cancer-derived exosomes [190]. Their methodology was based on generating a shear force on functionalized-antibody surfaces of nanoelectrodes, which improved the specificity to capture targeted exosomes without interfering with other types of similar-sized vesicles. One year later, two microfluidic chips were fabricated and connected by a third independent research group (taller research group). These were based on surface acoustic wave and ion exchange concepts. First, exosomes were lysed at a rate of 38% using the surface acoustic wave; then, hsa-miR-550 was detected in pancreatic cancer cell lines to a limit of two picomolars using ion-exchange sensing. These connected chips were time- and sample-saving [171]. Ramshani and coauthors improved on Taller′s device in 2019. Their device had an electrokinetic membrane sensor based on nonequilibrium ionic currents [74], and they detected and quantified miRNA-21 in generated liver cancer plasma within 30 min. Their device was (1) reproducible for other biomarkers and (2) did not require sample pretreatments.

Daaboul and coauthors (2016) presented a new method for characterizing exosome phenotypes based on their sizes. Their microarray chip allows the automatic quantification of the sizes of individual exosomes >50 nm using an interferometric reflectance imaging sensor [14]. Their chip was simple and had a high sensitivity for low sample volumes (20 µL). In the same year, Etayash and his research team reported a cantilever array for simultaneously detecting overexpressed membrane proteins CD24, CD63, and EGFR in exosomes derived from breast cancer [163]. Zhao and his team introduced an additional chip to offer a continuous-flow ExoSearch platform for exosomes derived from ovarian cancer. The ExoSearch chip performs a continuous quantification of exosomes in blood plasma sample volumes from 10 μL to 10 mL using immune-magnetic beads to detect three biomarkers: CA-125, EpCAM, andCD24 [110].

To overcome the challenges of exosome-based methods for cancer diagnosis, such as (1) low density, (2) tiny size, (3) difficulty of isolation from plasma, (4) lab-consuming steps, and (5) enrichment and purification issues [116,191], an electrokinetic microarray chip was fabricated [192]. This chip provides several advantages, such as (1) a low sample volume (30−50 μL), (2) time savings—the process takes less than 30 min, (2) enablement of subsequent on-chip immunofluorescence detection of exosomal proteins, and (3) provision of viable mRNA for RT-PCR analysis [192]. A simultaneous readout device was fabricated. This device was based on peroxidase substrate 3,3′,5,5′-tetramethylbenzidine (TMB) oxidation catalyzed by an exosome–antibody complex [193]. This device exhibited (1) a high-throughput analysis, (2) a low detection limit—2.7 × 10^3^ particles/µL, and (3) rapid detection without sophisticated instruments [193].

Additionally, a droplet-based enzyme-linked immunosorbent assay (ELISA) chip was fabricated to quantify cancer-derived exosomes in low sample volumes with low concentrations [194]. First, the exosomes were captured on magnetic beads through enzymatic ELISA complexes; then, the bead/exosome complexes were encapsulated inside the droplets and counted [194]. This improved the isolation, detection, and quantification methodology. A well-organized 16 × 20 × 20 pillar brush-like organized structure was fabricated for real-time accommodating, imaging, and detecting of single exosomes. Each pillar was capped with an 80-nm gold cap conjugated with anti-CD63. This amazing chip was fabricated to detect breast cancer cells (MCF7) [69]. Zhang and coauthors presented a developed chip integrated with 3D herringbone structures to detect exosomes at concentrations as low as 10 particles/μL, which were undetectable by standard microfluidics. They detected exosomes derived from ovarian cancer and normal exosomes in 20 and 10 samples, respectively. CD24, EpCAM, and FRalpha proteins were used as biomarkers. Their chip boasts (1) a large surface area and (2) high exosome-binding efficiency [143].

In 2019, a label-free microarray was developed to detect exosomes released from macrophages to evaluate the immune response. The chip had seven exosomal-specific antibodies fabricated on a photonic crystal biosensor surface. This chip (1) was cost-effective, (2) fast, and (3) required a low sample volume of 1 μL [111].

#### 4.2.3. Specific Raman Scattering Technology

**Figure 7:**
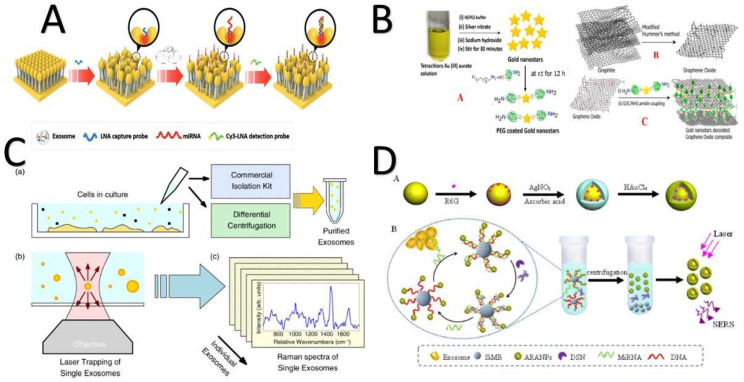
Examples of Raman scattering techniques for exosome detection and quantification. The figure permissions are as follows: (**A**) license number 5071621135225, license date May 17, 2021, licensed content publisher John Wiley and Sons. Licensed content publication of “Quantitative and Specific Detection of Exosomal miRNAs for Accurate Diagnosis of Breast Cancer Using a Surface-Enhanced Raman Scattering Sensor Based on Plasmonic Head-Flocked Gold Nanopillars”. (**B**) Adapted with permission from Pramanik, A. et al. 2020. Mixed-dimensional heterostructure material-based SERS for trace level identification of breast cancer-derived exosomes. ACS omega, 5(27), pp. 16602–16611. Copyright (2021), hps://pubs.acs.org/doi/10.1021/acsomega.0c01441, and further permissions related to the excerpted material should be directed to ACS. (**C**) Adapted from Smith, Z.J. et al. 2015. Single exosome study reveals subpopulations distributed among cell lines with variability related to membrane content. *Journal of extracellular vesicles*, 4(1), p. 28533. The work was made available under the Creative Commons CC0 public domain dedication 10/2021 (http://creativecommons.org/licenses/by-nc/4.0/). (**D**) License number 5071700114394, license date 17 May 2021, licensed content publisher Elsevier. Licensed content publication of “Quantitative detection of exosomal microRNA extracted from human blood based on surface-enhanced Raman scattering”.

Specific Raman scattering (SRS) technology has received a great deal of attention because of its ultra-sensitivity in the detection of a variety of small biological fractions with low background noise [112] (Figure 7). The Ma research group proposed using miRNA-mediated gold–silver nanoparticles with internal nanogaps to detect miRNA-21 in non-small-cell lung cancer (NSCLC). First, an Au-rhodamine 6 G (R6G)–AuAg complex with internal nanogaps was prepared, which was then attached with silicon microbeads to the 3′- and 5′-ends of the capture probe. The capture probe targeted miRNA-21 exosomes. A duplex-specific nuclease cleaved the exosomal miRNA to release SRS signals from the surface of the Au complex. However, the miRNA-21 was still in contact and cycling the amplification to release more Au complexes. The solution intensity was directly proportional to the concentration of exosomal miRNA. This method introduced a new technique for quantifying specific exosomal miRNA in low sample volumes (5.0 μL) for clinical purposes [113].

A similar approach using a different functionalized gold (Au) surface to target and quantify exosome MicroRNA-10b as a biomarker for pancreatic cells was reported. The iron (III) oxide/silver/DNA/Au–silver/ DTNB (5,5-dithio-bis-(2-nitrobenzoic acid) complex was able to detect miRNA-10b in blood samples and differentiate among pancreatic cancer, chronic pancreatitis, and healthy samples [172]. Another method for the reliable detection of miRNA was proposed in 2019. Chen and his research group invented a specific Raman scattering (SERS) platform to detect the presence of miRNA-21 where miRNA-21 was a key to triggering the allosteric effects of mismatched catalytic hairpin assembly (CHA) amplification. The hairpin H1 probe opened and hybridized with the hairpin H2 probe to form a H1–H2 complex. This complex combined with DNA on the enzyme-free surface of the platform and signals were recorded. 4-aminothiophenol was the internal standard, coupled with CHA. Their platform exhibited a sensitivity range from 10 fM to 100 nM [195].

Coupling the magnetic nanobead capturing technology with SRS, a developed SRS was investigated for recognizing and detecting CD63 as a general cancer biomarker. The developed SRS was composed of modified gold shell magnetic nanobeads for capturing purposes, three different gold nanoparticle probes for the instant detection of most kinds of exosomes, and a Raman reporter for signal amplification. In a solution with a target exosome, an exosome/magnetic beads-appropriate probe complex was formed, and the signal indicated the presence of target exosomes [158].

Another Raman scattering platform for quantifying the exosomal miRNA derived from breast cancer cells was developed by Lee and coauthors [196]. It was characterized by multiple spots on gold-coated nanopillars and a capability for hybridization between small oligonucleotides, e.g., miRNAs, and locked nucleic acid probes. The design offers (1) a low detection limit, 1 am–100 nm, (2) miRNA recovery, and (3) multisensing opportunities [196]. One year later, an enhanced hetero-structured (2D-0D) Raman scattering spectroscopy was proposed for tracking exosomal HER^2+^ derived from breast cancer cells [160]. The authors applied physical and chemical enhancement mechanisms. Physical enhancement was applied via the electromagnetic field produced by the plasmonic gold nanostar. Chemical enhancement was applied via the presence of 2D graphene oxide material. This platform was able to track exosomes to a limited concentration of 4.4 × 10^2^ particles/mL [160].

As mentioned above, the Ma research group [113] proposed using miRNA-mediated gold–silver nanoparticles with internal nanogaps to identify NSCLC-derived exosomes using miRNA21. Pang et al. [197] used exosomal PD-L1 in a 40-min test for the same purpose, with 96% efficiency. First, the hydrophilic heads of the exosome bilayer phospholipids bind to the TiO_2_-–Fe_3_O_4_ complex and are directly separated from the serum. Afterwards, the exosomal PD-L1 is labeled when binding to the anti-PD-L1 antibody-modified silver–gold mercaptobenzoic acid (MBA) SRS tags [197]. Ning et al. [198] developed a gold–silver bimetallic nanotrepang-based SRS biosensor for monitoring cancer-derived exosomes. The probes were composed of core nanorod gold–silver shell/silver shell active nanotags decorated with DNA linkers. Three probes with modified magnetic beads were used to capture exosomes with PSMA, HER^2+^, and AFP biomarkers to identify prostate cancer, breast cancer, and hepatic cancer cell lines, respectively. After capturing target exosomes, the nanotags released them inside the solution, and the appropriate signals were detected on the magnetic bead surfaces. This biosensor offers reliability with multi-biomarker exosome detection [198].

#### 4.2.4. Electrochemical Techniques

**Figure 8:**
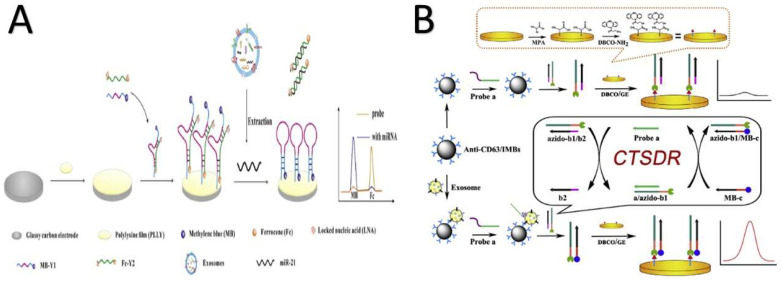
Examples of electrochemical platforms for exosome detection and quantification. The figure permissions are as follows: (**A**) license ID 1147246-1, license date 13 September 2021, licensed content publisher ELSEVIER BV. Licensed content publication of “A ratiometric electrochemical DNA biosensor for detection of exosomal MicroRNA”. Copyright Clearance Center, Inc. (CCC) grants licenses on behalf of the rightsholder (Royal Society of Chemistry). (**B**) License ID 1120257-1, license date 13 September 2021, licensed content publisher Pergamon. Licensed content publication of “A catalytic molecule machine-driven biosensing method for amplified electrochemical detection of exosomes”. Copyright Clearance Center, Inc. (CCC) grants licenses on behalf of the rightsholder (Royal Society of Chemistry).

Electrochemical systems have emerged as a solution to the challenges of miRNA: (1) miRNA has short base pairs (19–25 base pairs), (2) it is naturally occurring in very low concentrations, (3) it has sequence similarity, (4) it is labor-intensive, (5) it has an amplification bias, and 6) it is expensive to manipulate [19,30,199,200]. Several electrochemical systems were designed to detect cancer-derived exosomal biomarkers derived from human samples [121] (Figure 8). All electrochemical methods possess certain characteristic advantages: (1) high sensitivity, (2) ease of handling, (3) time savings, (4) use of nontoxic materials, (5) low background, and (6) simple instrumentation [199,201,202].

In 2016, Zhou et al. developed an aptamer-based electrochemical microfluidic chip to detect and quantify CD63, the cancer biomarker [7]. Their system relies on the electrochemical potential signal decreasing because of prelabeled strands from the functionalized surface of a gold electrode being released when the exosomes are captured by anti-CD63 beads. Their system offers (1) a low detection limit, 10^6^ particles/mL, which is 100-fold less than those of known commercial kits at this time, and (2) direct detection with no need for pretreatments. In 2017, Smith et al., researchers in the UK, reported a pioneering electrochemical biosensor with a functionalized glass carbon electrode for specifically identifying miR-21 in urine for prostate and bladder malignancy patients. This pioneering system offers a much lower detection limit, 20 × 10^−15^ molar [147]. Tavallaie and coauthors designed a novel reconfigurable DNA–Au@MNP electrical network for the direct detection of miRNA concentrations of 10 aM–1 nM in untreated blood samples [203].

In 2018, another research group in China created an ultrasensitive ratiometric electrochemical biosensor for miRNA-21 in breast cancer cells. Their biosensor applied DNA walkers, DNA tracks, a target–response reporter, and a reference reporter. Applying these features offers advantages such as (1) low cost; (2) reproducibility, as it can be used up to five times, (3) high selectivity, and (4) a low detection limit, 67 aM [162]. Assembling the ratiometric electrochemical biosensor, Zhao et al. [164] and Wang et al. [204] developed their electrochemical biosensors to selectively detect cancer-derived exosomes. Zhao et al. applied their biosensor to the detection of exosome DNA captured with anti-CD63 and EpCAM aptamers derived from breast cancer cells. Zhao used a 3D DNA walker and an exonuclease-III-assisted electrochemical ratiometric sensor to reach a detection limit of 1.3 × 10^4^ particles/mL [164]. Wang applied cholesterol-labeled DNA strands to plug into the bilayer of captured exosomes, with a detection limit of 29 particles/µL [204]. Cao and coauthors designed an electrochemical cell to detect exosomes derived from hepatic cancer. The detection part has a CD63 aptamer combined with a DNA chain combined with immunobeads functionalized with anti-CD63. This method possesses a high specificity level with a low detection limit, 1.72 × 10^4^ particles/mL, which provides adequate details on cancer prognosis monitoring [205]. Boriachek and coworkers selectively isolated exosomal miRNA from eight serum samples on a surface of pre-functionalized magnetic beads, which then automatically adsorbed on a gold electrode. The adsorbed miRNA was electrochemically detected in the presence of a redox system ([Fe (CN)_6_]^4−/3−^) with a sensitivity of 1.0 pM [121].

One year later, in 2019, Huang and coauthors invented a label-free electrochemical aptasensor to specifically detect exosomes derived from gastric cancer exosomes. Again, anti-CD63 loaded onto a gold electrode surface was used to capture exosomes. Then, circle amplification was triggered as a response of the exosome capture step, and multiple G-quadruplex units were produced. An electrochemical signal accumulated when the hydrogen peroxide was reduced, and horseradish peroxidase mimicking DNAzyme acted as a catalyst. This aptasensor is specific and sensitive, as its detection limit is 9.54 × 10^2^/mL [175]. Continuing with the same concepts, Qiao and his team reported a system for identifying exosomal CD63 derived from breast cancer cells from serum samples. They used mercaptopropionic acid (MPA)-modified Eu^3+^-doped CdS nanocrystals as the electrochemiluminescent emitters and hydrogen peroxide as the core actant. After recognizing the exosomes, the H_2_O_2_ decomposed, and the electrochemiluminescence (ECL) signals decreased [206]. The limit of detection (7.41 × 10^4^ particles/mL) was twice as low as that of the Rongrong design [175]. Other efforts produced a CD63-fuctionalized reduced graphene oxide field effect transistor biosensor to quantify cancer-derived exosomes in low-concentration samples (33 particles/µL) electrically. This device was capable of distinguishing prostate cancer samples from control samples without labeling [11].

A peptide nucleic acid (±)/microRNA/spherical nucleic acid nanoprobe (+) sandwich on an electrochemical sensor was developed by the Liu research team to detect exosomal miRNA in blood samples from breast cancer patients. This sandwich sensor was (1) specific because of the hybridization opportunity between the neutral and the negative sides of the sandwich, which enhanced the detection process, (2) sensitive enough to detect a single base mismatch, (3) label-free, and (4) an enzyme-free process [207]. Recently a sandwich structure for diagnosing ovarian malignancy early by detecting phosphatidylserine (PS)-positive exosomes was created by Liu. Exosomes were captured on the surfaces of functionalized gold nanoflowers that capped g–C_3_N_4_ nanosheets. When exosomes were captured, the nanosheets catalyzed the decomposition of H_2_O_2_, and the ECL signals were amplified. In addition to its sensitivity and low cost, this system possesses the low detection limit of 39 particles/µL [146].

Applying 2D Ti_3_C_2_ MXenes nanosheets and anti-EpCAM as the ECL probe and the capture aptamer, Zhang designed a reliable, sensitive biosensor to detect breast cancer in serum samples with a detection limit of 125 particles/µL [137]. A novel ECL system conjugated with polymerase amplification was constructed to detect miRNA-16 derived from leukemia [141]. A magnet-controlled glassy carbon electrode functionalized with a nano-Au surface that adsorbs assistance DNA with the pyridine–ruthenium complex included the chemical components of the system. When the target miRNA was detected, the capture DNA immobilized on the electrode opened and was conjugated with that target to form double-stranded DNA. Then, the hairpin structure opened, and the DNA primers conjugated with the complementary sequence occurring at the hairpin neck with the assistance of the Klenow fragment of DNA polymerase and the ECL signals were recorded. This biosensor is (1) label-free, (2) ultrasensitive, with the low detection limit of 4.3 × 10^−17^ mol/L, (3) stable, and (4) reproducible [141].

In 2020, an electrochemical biosensor for exosomal microRNA detection was realized that has two steps: (1) the induction of miRNA signal amplification and (2) induction of silver nanoparticle deposition. This method is simple, inexpensive, and ultrasensitive (limit of 0.4 fM) [200]. An improved electrochemical system for exosome detection was fabricated by Fang and coauthors. They enhanced their system by combining the benefits of (1) MXenes, which act as a probe supporter to improve the signals, (2) black phosphorous quantum dots (BPQDs), which act as an oxidation catalyst for tris (4,4′-dicarboxylicacid-2,2′-bipyridyl) ruthenium (II) dichloride (Ru(dcbpy)_3_)^2+^ and also improves signals, and (3) SiO_2_ nanourchin, which acts as a sensing platform for the aptamer. Both the MXenes and the BPQDs provide extraordinary photothermal properties. Their systems operate in two forward steps: First, EpCAM is detected on an immobilized aptamer; second, the modified exosomes are combined with the CD63 antibody on the MXenes-BPQDs–Ru(dcbpy_3_)^2+^ complex [115]. This dual ECL system is specific and effective. Another electrochemical assay was fabricated and hybridized with the chain reaction method to detect miR-122 in liver and breast cancers [167]. The presence of miR-122 opens hairpin DNA immobilized on a gold electrode surface and triggers a hybridization chain reaction to generate long double helixes that capture more [Ru (NH_3_)_6_]^3+^ and increases the differential pulse voltammetry. The structure and controlling features of this method offer a promising amplification efficiency and high sensitivity. A self-reductant ECL biosensor was developed to derive benefits from the hybridization of Ti_3_C_2_–MXenes/gold nanoparticle complexes and CD63 aptamer to capture and detect tumor-derived exosomes. This naked catalytic surface biosensor delivers high sensitivity, excellent conductivity, a large surface area, and the low detection limit of 30 particles/μL, which is 1000-fold lower than that of ELISA [208]. A ratiometric electrochemical DNA biosensor was also fabricated and modified with a Y-shaped locked nucleic acid to detect exosome miR-21 derived from breast cancer. This modification increased the selectivity and accuracy and brought the sensitivity to a detection limit of 2.3 × 10^−15^ moles [209].

Human epidermal growth factor receptor (EGFR) and the EGFR variant (v) III mutation (EGFRvIII) are exosomal biomarkers that have been used for the early detection of fatal brain tumor glioblastoma from circulating blood. In the designed electrochemical system, (1) a peptide ligand binds to the EGFR and EGFRvIII, (2) Zr^4+^ from the metal organic framework/electroactive methylene blue complex binds to the intrinsic phosphate groups on exosome surfaces, and (3) the exosome concentrations are quantified directly from the concentration of electroactive molecules on the exosome surfaces. The detection limit is 7.83 × 10^3^ particles/μL [173].

Applying the strategies of locked nucleic acids, a G-quadruplex, and rolling circle amplification and using a gold electrode, Xiaoqi and coauthors designed an electrochemical system to detect exosomal miRNA-21 with a detection limit of 2.75 × 10^−15^ M. Their design was comparable to the RT-PCR strategy because of its stability, consistency, and reproducibility [210].

A dual-signal self-calibrating biosensor for detecting cancer-derived exosomes was developed from previous electrochemical protocols [211]. The authors designed their platform using black phosphorus nanosheets associated with a metal–organic framework, which hybridized with antibody thin films to capture exosome biomarkers. Both the nanosheets and the framework were assembled on an indium tin oxide slice, which was attached to a ssDNA aptamer labeled with methylene blue. The redox current of the methylene blue was reduced in the presence of the desired exosomes. This workstation offered a low detection limit—100 particles/mL, specificity, and the capability of detecting multiple biomarkers [211].

Meng and colleagues designed a novel on/off photoelectrochemical biosensor applying an organic dye (toluidine blue O dye) to detect cancer biomarker miRNA 21. A low concentration of miRNA 21 (10^17^–10^11^ mol/L) could be detected when transformed into abundant p-type copper sulfide (CuS)-labeled signal tags and combined with TBO@Bi_2_S_3_-ZnS. The photoelectrochemical signals could then be used to detect and quantify miRNA 21 [212].

Electrochemical techniques, which have been the subject of research efforts since 2016, have many applications and are considered the most promising techniques for exosome detection and cancer diagnosis, because all the electrochemical techniques are easy to handle, rely on nontoxic materials, are timesaving, and have high sensitivity with a low background.

#### 4.2.5. Fluorescence and Colorimetric Techniques

**Figure 9:**
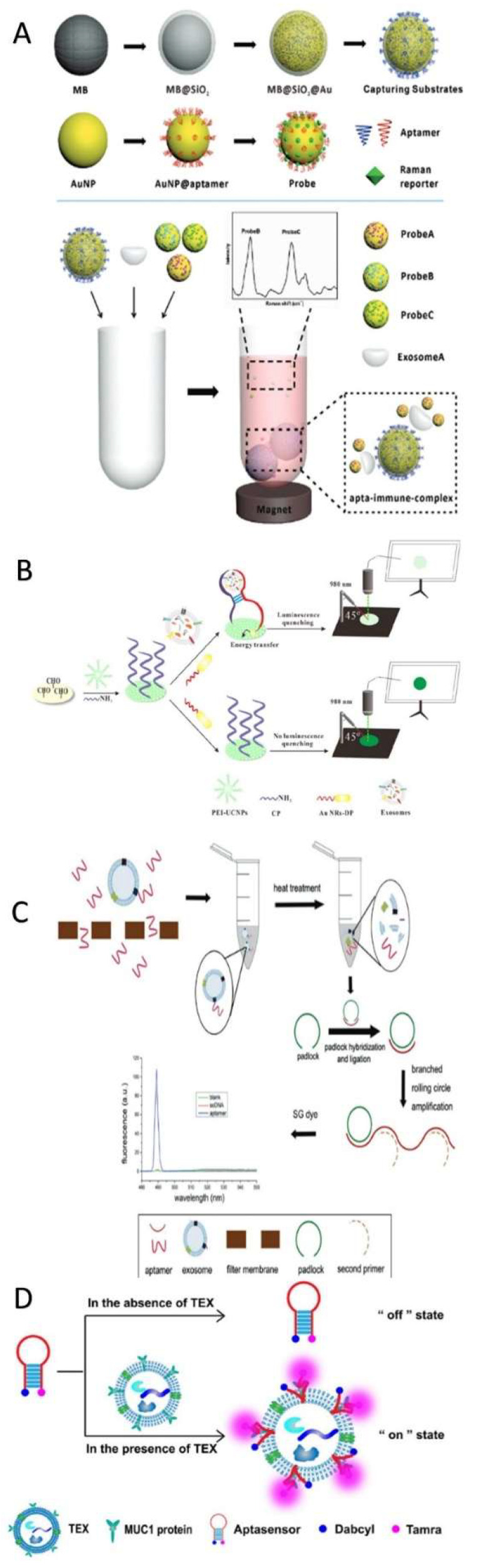
Examples of fluorescence techniques for exosome detection and quantification. The figure permissions are as follows: (**A**) license ID 1119684-1, license date 17 May 2021, licensed content publisher RSC Pub. Licensed content publication of “Screening and multiple detection of cancer exosomes using an SERS-based method”. Copyright Clearance Center, Inc. (CCC) grants licenses on behalf of the rightsholder (Royal Society of Chemistry). (**B**) License ID 1147245-1, license date 13 September 2021, licensed content publisher Pergamon. Licensed content publication of “A paper-supported aptasensor based on upconversion luminescence resonance energy transfer for the accessible determination of exosomes”. Copyright Clearance Center, Inc. (CCC) grants licenses on behalf of the rightsholder (Royal Society of Chemistry). (**C**) License ID 1119685-1, license date 17 May 2021, licensed content publisher RSC Pub. Licensed content publication of “A simple fluorescence aptasensor for gastric cancer exosome detection based on branched rolling circle amplification”. Copyright Clearance Center, Inc. (CCC) grants licenses on behalf of the rightsholder (Royal Society of Chemistry). (**D**) License Number 5071720075356, license date 18 May 2021, licensed content publisher Elsevier. Licensed content publication of “A simple, specific and “on-off” type MUC1 fluorescence aptasensor based on exosomes for detection of breast cancer”.

The main differences between fluorescence immunoassays and colorimetric techniques are the high sensitivity and accuracy of the fluorescence immunoassays [213]. Here, we focused on the published designs (37 models) using either one of the methods to detect/quantify cancer-derived exosomes for diagnostic purposes (Figure 9).

As miRNAs are responsible for regulating gene expression and are protected from ribonuclease degradation, they are excellent biomarkers for the diagnosis and prognosis of cancer [195,199,200]. In 2008, Taylor and Gercel-Taylor were able to separate exosomes derived from ovarian cancer from serum samples by applying a modified magnetically activated cell sorting technique based on identifying the exosomal EpCAM protein using a LD microcolumn. The exosomes and beads were separated, and the exosomes were recovered in PBS by ultracentrifugation. The miRNAs were isolated using a mirVanamicroRNA isolation kit, then labeled with Cy3, hybridized using microarrays, and scanned with an array scanner. Perfect matches for miR-27a, miR-93, and miR-152 plus two mismatches for each one was applied. According to Taylor’s procedure, detecting certain miRNAs (miR-21, miR-141, miR-200a, miR-200c, miR-200b, miR-203, miR-205, and miR-214) are the key to discriminating between ovarian cancer samples and benign disease or healthy samples [18].

Ovarian cancer diagnosis research has been extended to screening exosome claudin proteins, e.g., claudin-4, as biomarkers. In 2009, Li et al. found full-length claudin-4 shed from a plasma culture in a conditioned culture medium following immunoblotting. They detected it using sucrose gradient separation and immunogold electron microscopy experiments. The authors presented their procedure as a step for confirming ovarian cancer detection combined with further analysis [145]. Additionally, in 2009, Logozzi presented a novel tool for screening for and following-up on melanoma-derived exosomes. Logozzi invented a home-based sandwich ELISA to capture exosomes CD63 and Rab-5b and to quantify caveolin-1 from mice plasma samples as follows: (1) isolate exosomes using ultracentrifugation, (2) coat 96-well plates with anti-Rab-5b, (3) incubate exosomes overnight inside the functionalized 96-well plates, (4) add anti-CD63 Mab or anticaveolin-1 Mab, and (5) record the density at 450 nm. The results collected from the invented tool were confirmed using Western blot and flow cytometry. Melanoma samples: *n* = 0 compared to healthy cases: *n* = 58 [178].

By 2015, several research efforts were published. It was found that exosome c-Met promotes the progression of melanoma [214]. A fast, simple, and sensitive procedure for capturing, detecting, and quantifying exosomal miRNA-21 from exosomes derived from breast cancer was reported. Simply by functionalizing molecular beads with streptolysin O and fluorescent dye Cys3, Lee and his team were able to selectively identify and quantify miRNA-21 mixed into human serum [20].

Melanoma-derived exosomes inside lymph nodes pulse signals to control cell recruitment, the spread of vascular tissues inside the lymph nodes, and the deposition of fluids [215]. For the first time, melanoma exosomes were imaged in vitro and within lymph nodes in vivo, using magnetic resonance imaging to prove those exosomes can be monitored in vivo. The exosomes can be tracked after they are loaded onto superparamagnetic iron oxide nanoparticles [194].

To report the progression of tumors implanted in mice, microtoroid optical resonators were applied. The target exosomes were only able to land on a functionalized silica microtoroid and change its resonance frequency. This method was label-free, applicable to a range of exosome sizes, and sensitive [216].

In 2016, Ahadi and coauthors published a dataset identifying the long noncoding RNA exosomes related to prostate cancer. After testing the exosomes from five cell lines, they concluded that there was a significant expression difference between the exosomes and their original cells [217]. They found that miR-17, miR-18a, miR-20a, miR-93, miR-106b, and thelet-7 were highly expressed in prostate cancer samples. A rapid, one-step, and novel flow immunoassay strip was invented in 2016 for capturing CD9 and CD81, detecting CD63 labeled with nano-Au, and quantifying exosomes derived from melanoma cancer in plasma and urine samples based on the use of tetraspanins as targets [179]. In addition, Kibria et al. (2016) were able to distinguish a single circulating exosome biomarker in human cell lines and blood samples using a microflow cytometer. This captured exosome, CD63, then applied fast staining and automated counting steps. They were also able to compare the expression of CD44 and CD47 between breast cancer and healthy samples [6]. miR 182, which is dose-independent in breast cancer, was detected in both the cell culture supernatant and fresh serum. Higginbotham and coauthors developed a fluorescence-activated vesicle-sorting platform for detecting and sorting individual exosomes from cell lines based on specific exosome-surface biomarkers. Their protocol is based on (1) sequential ultracentrifugation to isolate exosomes, (2) the capturing and labeling of exosomes using labeled fluorescent antibodies, and (3) the detection of individual exosomes using a commercial flow cytometer. They were able to detect EGFR and CD9 as biomarkers for human colorectal cancer and recognized the activation state of EGFR using monoclonal antibody 806 [218]. A capillary electrophoresis/mass spectrometry method was applied to isolate and analyze iso-miR-16-5p and miR-21-5p in serum samples from leukemia patients [114].

In 2017, for the first time, carcinoembryonic antigen was detected and quantified in colorectal cancer-derived exosomes in serum. At Wakayama Medical University Hospital, 116 patients were tested through one year using an ELISA protocol, and the protocol was optimized [219]. In the same year, single-stranded DNA was found to improve the peroxidase activity of g–C_3_N_4_ nanosheets, yielding a four-fold increase in the colorimetric analysis of exosomal CD63 in breast cancer cells. The reason for this was the electrostatic attraction and aromatic stacking between the DNA and the nanosheets [220]. An on-off aptamer was designed to detect MUC1 on breast cancer-derived exosomes. The aptasensor had two ends, one representing TAMRA (luminophore) and the other representing Dabcyl (quenching group). In the presence of exosomes, the aptasensor was locked, and MUCI was recognized by the aptasensor, which turned on and emitted fluorescence signals. This protocol offers applicability, sensitivity, and specificity [107].

Three microchip and electrochemical technologies allowing the detection of exosomes with the naked eye were mentioned above [190,193,208]. Other published platforms allowing detection with the naked eye applied calorimetric and fluorescence technologies. The first one was designed by Reference [151]. The mechanism was based on substituting the aptamer/Au nanoparticle complex with aptamer/exosome surface proteins and releasing Au nanoparticles, which aggregate in the ultra-salty solution and change the solution color [151]. Additionally, Zhang and his team initiated a novel protocol for virtually recognizing cancer-derived exosomes. Their protocol was based on the following steps: (1) capture exosomes through binding with anti-CD63 on a magnetic bead aptamer, and (2) immediately insert a modified DNA probe into the exosome membrane to trigger the hybridization chain reaction, transduce the signal, and amplify the signal through alkaline phosphatase. As a result, silver ions are reduced by ascorbic acid and form a shell around gold nanoparticles, causing a vivid color change. This protocol offered limits of detection of 1.6 × 10^2^ particles/μL with UV−Visible spectroscopy and 9 × 10^3^ particles/μL with the naked eye [221]. The imaging protocol recently designed by Zhou [156] targets simplicity, rapidity, and low cost and can identify exosomes derived from breast cancer. This protocol, like the previous protocol published by Reference [107], is aimed at creating an on–off switch with a low limit of detection, 3.9 × 10^5^ particles/mL. The two ends of the aptasensor represent MUC1 aptamer as a luminophore and mimicking DNAzyme in a hairpin structure as a quenching group. If there are exosomes, they bind to the MUC1 aptamer; then, the DNA hairpin unfolds and triggers the conformation of G-quadruplex–DNAzyme. As a result, a clear blue color originates from the reduction of hydrogen peroxide [156].

Efforts have been made to fabricate a self-referenceable platform specifically for detecting and quantifying cancer-derived exosomal biomarker CD63. Researchers constructed a Cy3–anti-CD63 aptamer that adsorbed on Ti_3_C_2_ MXene nanosheets via metal interactions and hydrogen bonds. In the presence of the desired exosomes, the aptamer highly bonded with exosome CD63 released from the nanosheet surface and produced fluorescence signals that were referenced through the fluorescence resonance energy transfer nanoprobe. This self-referenced platform can be applied to detect several biomarkers at once, and it offers a limited range of detection, 1000-fold lower than that of ELISA [222]. Since the expression profiles of exosomal miRNA make them potential candidates for cancer diagnosis, Zhai and coauthors developed a 4-h detection and quantification protocol for detecting miR-1246 and identifying breast cancer samples in plasma. They constructed a functionalized Au nanoflare probe with nucleic acids that were able to enter the exosomal plasma and generate fluorescence signals. This in situ detection protocol is (1) inexpensive, (2) fast, (3) simple, and (4) specific [24]. Another miRNA was investigated as a biomarker for ductal carcinoma, which is sometimes a result of breast cancer. Yoshikawa investigated miR-223-3p as a biomarker for diagnosing ductal carcinoma in 185 plasma samples using a miRNA microarray and TaqMan miR assays [177]. Both CD63 and nucleolin were used as targets to capture exosomes derived from leukemia cells using anti-CD63 antibody conjugated with magnetic beads. After that, a DNA primer–nucleolin–recognition aptamer bound the exosomes, initiating the amplification reaction and generating gold nanoparticle/DNA/fluorescent dye complexes. Through the help of nicking endonuclease, the fluorescent dye was released, emission started, and signals accumulated [142]. This dual fluorescence platform provides sensitivity for sample concentrations as low as 1 × 10^2^ particles/μL. Another fluorescent biosensor for detecting miRNA-21 was fabricated by Reference [148]. This biosensor couples the target-catalyzing signal amplification with DNA-labeled carbon dots and a 5,7-dinitro-2-sulfo-acridone probe to reach high fluorescence resonance energy transfer when the probe is assembled. This ratiometric biosensor is (1) sensitive, with the low detection limit of 3 × 10^−15^ M, (2) stable, (3) capable of removing environmental fluctuations, (4) selective of a single base mismatch sequence, (5) cost-effective, and (6) usable for monitoring purposes [148].

In 2019, a direct method for exosomal DNA detection was published. When exosomes were captured on the CD63 aptamer surface, the DNA probe initiated a hairpin DNA cascade reaction, and the open DNA hairpin bound to gold nanoparticles. The signals of the labeled DNA dendrimers were recorded. The limit of detection was 1.16 × 10^3^ particles/μL [223].

Another way to overcome the disadvantages of exosomal miRNA detection for early cancer diagnosis and monitoring is to combine the detection of the miRNA pool with surface proteins to reflect the tumor heterogeneity. Cho and coworkers (2019) found that miR-21, miR-574-3p, EpCAM, and epidermal growth factor receptor (EGFR) were more highly expressed in exosomal cancer-derived cells than in noncancer cells. Exosomes derived from a prostate cancer biopsy were first isolated using anti-CD63 antibody. Then their miR-21, miR-574-3p, and EpCAM surface proteins were simultaneously detected and quantified in a single step using CD63/antibody/nanomolecular beacon/oligonucleotide probes at 37 °C for 60 min after normalization [19]. This multitarget detection method is (1) simple, (2) inexpensive, and (3) labor-saving. Like Cho, Shi proposed a protocol using magnetic beads, antibodies, and aptamer hybridized with chain reaction probes to detect exosomes derived from hepatic carcinoma at a limiting rate of 100 particles/mL. Three types of antibodies were used: prostate-specific antigen, D-dimer, and anti-CD63. This protocol requires the pretreatment of exosomes before manipulation [224].

Turning their attention from the miRNA biomarkers to other RNAs to use as biomarkers, researchers in Japan investigated the possibility of relating the expression of four mRNAs and five small nucleolar RNAs for the early diagnosis of pancreatic cancer. They found that the mRNAs (ARF6 and WASF2) and snRNAs (SNORA25 and SNORA74A) were highly expressed in patient samples and could be used as biomarkers [170].

Another on/off detector has been designed in which an afterglow semiconducting polyelectrolyte switches between the on and off modes according to the presence of target exosomes [225]. Different exosome proteins can be detected using different aptamer sequences. This design offers a detection limit that is twice as low as that of other fluorescence detection methods.

Recently, the Huang research group developed an assay specifically for detecting exosomes derived from gastric cancer. The desired exosomes bind to the aptamer, and branched rolling circle amplification is triggered when a second primer is added. SYBR Green I fluorescent dye is used to detect long, double-stranded DNA. This assay possesses throughput specificity, a detection limit of 4.27 × 10^4^ particles/mL, and a probability of an early diagnosis of gastric cancer [226].

A new colorimetric method for detecting exosomes derived from colorectal cancer patients (*n* = 16) and comparing them to healthy ones (*n* = 9) was invented by Huang et al. 2020. Their detection method was based on the enzymatic activity of deoxynucleotidyl transferase. Two probes were selected: anti-A33 as a capture probe and the EpCAM aptamer/Au/primer complex as a signal probe. After capturing exosomes, deoxynucleotidyl transferase helps the signal probe to reach the biotin adenine chains and bind them to avidin-modified horseradish peroxidase for the hydrogen peroxide-mediated oxidation of 3,3′,5,5′-tetramethyl benzidine in an enzyme-linked aptamer-sorbent assay (ELASA). The results showed high sensitivity, with the method detecting 1.95 × 10^6^ particles/μL. This platform also provides flexibility; the probes can be changed to detect different biomarkers [108]. In a new sandwich-type biosensor developed for cancer diagnosis, functionalized magnetic beads with anti-CD63, anti-EpCAM, and horseradish peroxidase catalysis were applied. After capturing the exosomes with anti-CD63, anti-EpCAM captured the exosomes derived from hepatic cancer. At this point, the oxidation–reduction reaction was triggered, and the fluorescent spectra at 370–550 nm were detected with a detection limit of 200 particles/mL [227].

A magnet-based immunoassay for characterizing and quantifying general and specific biomarkers for breast cancer patients was presented recently [155]. The exosomes were preconcentrated using anti-CD9, CD63, and CD-81-functionalized magnetic beads, then labeled with a second antibody for detecting CD24, CD44, CD54, CD326, and CD340. The second group of antibodies was conjugated with ELISA to quantify the signals. The detection limit of this procedure was 10^5^ particles/µL, and there was no need for pretreatments [155]. Immuno-translating exosomal proteins helps us to determine their structure and physiological roles. An immunosensor was developed to exploit CD63 on exosome surfaces. First, the desired exosomes are isolated using a size-exclusion chromatography. Then, the desired exosomes are detected using an anti-CD63-functionalized quartz crystal microbalance with dissipation monitoring. The detection limit of this technique (2.9 × 10^8^ particles/mL) is much higher than those of other techniques [228].

Xia and coauthors developed a cactus-like biosensor in which anti-CD63 mimicked the roots, captured exosomes mimicked the stem, and modified streptavidin/horseradish peroxidase/cholesterol-labeled DNA mimicked the thorns when inserted into an exosome membrane. The signals were generated from fluorescein because of the catalyzed oxidation of 1,4-phenylenediamine. This biosensor is comparable to known methods, because it is sensitive with no interference from proteins, has a detection limit of ~3 × 10^3^ particles/μL, and quantitatively measures the exosome extraction [229]. An advanced all-in-one biosensor was fabricated to simultaneously detect multiple biomarkers derived from breast cancer (miR-21, miR-27a, and miR-375) based on competitive strand displacement. This biosensor has three Y-type scaffold oligonucleotides, each of which has ss-recognition sequences associated with three quenchers and three labeled reporters conjugated with fluorophores FAM, Cy3, and Cy5. The protocol for this biosensor is based on intra-communication between the biosensor and the exosomes: the oligonucleotides complementarily hybridize with miRNA inside exosomes and release the reporter to activate the fluorescent signals. The limit of detection for this biosensor was found to vary according to the target miRNA and ranges from 0.116 to 0.287 μg/mL. This biosensor could be a routine bioassay for clinical purposes [166].

Fluorescence immunoassays and colorimetric techniques for detecting/quantifying cancer-derived exosomes are highly sensitive and easy to manipulate and have been upgraded to allow detection with the naked eye, which makes them fast and accurate to follow.

Many published research efforts have indicated the suitability of methods characterizing and detecting exosomes for procedures for monitoring cancer biogenesis. These methods include surface plasmonic biosensors; microchips; specific Raman scattering; and electrochemical, fluorescence, and colorimetric techniques. In addition, these methods can be used in applications that provide results that can be seen with the naked eye.

## 5. Conclusions and Future Prospects

Exosomes have been investigated as (1) disease biomarkers, (2) therapeutic agents, (3) activation motors, and (4) diagnostic tools. Exosomes are considered promising candidates for human disease and therapeutics biomarkers because they are easy to circulate through the blood, represent their parent cells, and are stable. Further exosome research is needed because of their heterogeneity and purification requirements and the need to attain supersensitive molecular profiling for individual exosomes with sizes of 30–50 nm [6,163,230].

Exosomes as disease-monitoring nanovesicles have been applied as rate-dependent biomarkers of disease stages [231]. We expect and suggest testing the informative role of exosomes as tools to predict the development of cancer diseases like Alzheimer’s disease. Certain exosome biomarkers (P-S396-tau, P-T181-tau, and Ab1–42) found to predict the development of Alzheimer’s disease up to 10 years in advance [232].

For another prospect, using exosomes to activate cells such as sperm [233] could be a start towards investigating the possibility of using exosomes as engines to activate immune-defense cells (T cells) and protect cells from cancer diseases. The future prospects for exosome research include, besides investigating exosomes for defense and prediction purposes, investigating the roles of exosomes derived from microorganisms such as bacteria and algae, as these organisms naturally produce multiple bioactive compounds.

## Figures and Tables

**Table 1:** Isolation techniques for exosomes.

Technique	Mechanism	Merits	Demerits	References
Ultracentrifugation	Isolation is dependent on the physical properties of the particles, and the density and viscosity of the solvent.	56% exosome isolation Easy to use Little or no sample pretreatment	Moderately time-consuming. Contamination and exosome losses	[12]
Ultrafiltration	Exosome isolation is depending on size fractions using specific membrane filters.	Fast No special equipment is needed Highest exosomal RNA yield. Automation, and scalability	Structure deformation and breakdown due to filtration force. Membrane lifetime because of clogging and vesicle trapping Errors due to exosome membrane attachment. Size exclusion limits	[71]
Size exclusion chromatography (SEC)	Sorting molecules according to their size on a porous stationary phase	Yields highly purified exosomes The structure, integrity, and biological activity are preserved Reproducibility	Special equipment is needed No scale up	[72]
Hydrostatic filtration dialysis (HFD)	Samples are forced through a dialysis tube using a low hydrostatic pressure.	Simple Effective Labor and cost-saving Scalable for large sample sizes	Time-consuming Size exclusion limits	[73]
Immunoaffinity capture–magneto-immuno-capture	This strategy to isolate culture-derived exosomes is based on magneto-immuno-capture	Fast, Easy Compatible with bench equipment The yield achieved was 10 to 15 times higher than that obtained by ultracentrifugation High capture efficiency Sensitivity Scalable for large sample sizes	Depending on sample concentration. Requires pretreatments	[74]
Mass spectrometric immunoassay	CD9 used as a general biomarker for that method	Highly specific techniques High RNA yield Requires smaller sample volumes (as little as 100 μL of sample compared to 2.5 mL used by ultracentrifugation)	Expensive equipment	[75]
Precipitation	Two-step process, incubation of the sample with the precipitation solution overnight at 4 °C, then isolation of the exosomes from the precipitate by either filtration or low-speed centrifugation	Easy No specialized equipment is needed Scalable for large sample sizes	Pretreatment is needed to remove cells and cellular debris. Contamination with molecules such as proteins and polymeric materials	[8]
Microfluidics	Different chips were designed to isolate exosomes according to their size and electromagnetic properties.	Rapidly and efficiently isolate exosomes Labor-saving	Scalability. Standardization. Some are time-consuming Some have low isolation efficiency.	[9]

**Table 2:** Physical and chemical characterization techniques for exosomal samples.

Technique	Target	Merits	Demerits	References
Transmission electron microscopy	Phenotype as shape and dimension	Direct method Requires a small sample amount	Expensive Sample preparation may lead to shape modifications	[97]
Nanoparticle tracking analysis	Size distribution and concentration	Fast No preparation steps	Inaccurate if samples are aggregated and/or have different size distributions, or the same instruments are in different geographical areas. Non specificity	[98]
Dynamic light scattering	Size distribution			[17,98]
Tunable resistive pulse sensing	Size distribution, concentration, and surface charge		Hard to select the appropriate nanopore setup	[99,100]
Atomic force microscopy	3-D topography	No fixation or staining steps Requires a small sample amount	Sample dehydration may lead to topography modifications	[101]
ELISA/Western Blot	Protein profile	Simple Specific Low sample volume	Sample preparation Time-consuming Inaccurate if detecting non-exosomal contents	[75]
Spectrophotometer	Protein and/or lipid concentration	Simple Fast No fixation or staining steps Requires a small sample amount	Sample preparation Inaccurate if detecting non-exosomal contents	[102]

**Table 3:** Detection techniques for exosomal samples.

Method	Approach Type	Target Component	Mechanism	Merits	Demerits	References
Surface plasmonic biosensor	Quantitative	Biomarkers	The electromagnetic field of surface plasmon, and the optical waves originate from the mass oscillations of electronic charge density of thin (nanoscale) metallic films	Integration, miniaturization, multiparameter, real-time, and label-free detection, Sensitivity	Not capable of identifying the post-transcriptional modifications of miRNA	[29,103,104]
Microchips-based Techniques	Quantitative	Various	Various designs according to the purpose and target	High-throughput for nonpurified samples Fast detection Easy to use, reagent-saving, and possessing high efficacy	Low mass transfer scale and interference with exosomal binding	[105,109,110,111]
Specific Raman Scattering Techniques	Quantitative	miRNA	Detection of captured exosomes with identified hairpins	Ultra-sensitive Low background noise.	Contamination issue	[112,113]
Electrochemical Techniques	Quantitative	Biomarkers	Decrease of the electrochemical signal because of the release of the pre-labeled stands from the functionalized surface of a gold electrode when the exosomes were captured by the anti-marker beads.	Reliable, fast Cost-effective Low sample concentration Sensitivity Easy to handle Saves time Nontoxic materials Low background, and simple instrumentation	Indict measurements	[75]
Fluorescent and Colorimetric Techniques	Qualitative/quantitative	miRNA	Label captured exosomes with stain e.g., Cy3	Fast, simple	Needs high sample concentration	[114]

**Table 4:** Biomarkers of specific proteins in exosomes according to disease type.

Disease Name	Exosome-Based Biomarkers	References
Colorectal cancer	A33, EpCAM	[108]
Leukemia	CD34	[140]
	miR-16	[141]
	CD63 and nucleolin	[142]
Ovarian cancer	CD24 and EpCAM FRalpha	[143]
	CA-125	[110,143,144]
	CA-125, EpCAM, CD24	[110]
	claudin proteins	[145]
	miR-21, miR-141, miR-200a, miR-200c, miR-200b, miR-203, miR-205, and miR-214) EpCAM-	[18]
	phosphatidylserine (PS)-positive exosomes	[146]
Prostate cancer	miR-21	[147,148]
	Survivin	[149]
	miR-1290 and miR-375	[122]
	PSA and PSMA	[150]
	PSMA	[151]
	miR-21, miR-574-3p, EpCAM, and epidermal growth factor receptor (EGFR)	[19]
	miR-17, miR-18a, miR-20a, miR-93, miR-106b and thelet-7family members	[152]
	PCA-3 and TMPRSS2:ERG	[153]
Prostate and breast cells	miR-183 family, which includes miRs-96, -182 and -183.	[154]
Breast cancer	CD24, CD44, CD54,CD326 and CD340	[155]
	mucin 1 (MUC1) protein	[107,156]
	HER2+	[157,158,159,160]
	miR-128	[161]
	miR-21	[20,162]
	CD47	[6]
	CD63	[69]
	CD24, CD63, and EGFR	[163]
	miR-1246	[24]
	CD63 aptamer and EpCAM aptamer	[164]
	miR-210	[63]
	2 exosome markers; CD9, CD63, 4 caner markers; CD24, CD44, EpCAM, and the human epidermal Growth factor receptor 2 (HER2)	[27]
	CD44	[165]
	CD44 and CD47	[6]
	miR-21, miR-27a and miR-375	[166]
Liver and breast cancer	miR-122	[167]
Hepatic carsinoma	AFP proteins	[168]
	(SMMC-7721)	[169]
Pancreatic cancer	sialylated Lewis (a) blood group antigen CA19-9	[170]
	hsa-miR-550	[171]
	MicroRNA-10b	[120,172]
Glioblastoma, (GBM), i one of the most fatal tumors in the brain	human epidermal growth factor receptor (EGFR) and EGFR variant (v) III mutation (EGFRvIII)	[173]
	CD63, and epidermal growth factor receptor variant-III	[174]
Gastric-cancer-derived exosomes	CD63	[175]
Lung cancer	miRNA-210	[176]
	EGFR, CEA, CYFRA 21-1, ENO1, NSE, CA 19-9, CA 125 and VEGF	[22]
	epidermal growth factor receptor (EGFR)	[29]
	miRNA-21	[113]
Invasive ductal carcinoma	microRNA-223-3p	[177]
Melanoma	CD63 and caveolin-1	[178]
	CD9 and CD81, detecting CD63	[179]
	melanocyte antigen A (MelanA)	[180,181]
Human epithelial colon cancer cells	Glycoprotein A33+ EpCAM+	[35,182]
Dendritic cells	MHC II+	[183]

**Table 5:** Biomarkers of specific proteins in exosomes according to cell type.

Cell Type	Biomarker Specific Protein	References
Human urine	ALIX (apoptosis-linked gene 2–interacting protein X) and TSG101 (tumor susceptibility gene 101 protein)	[184]
Human epithelial colon cancer cells	Glycoprotein A33+ EpCAM+	[35,182]
Dendritic cells	MHC II+	[183]
BT-474 breast cancer cells	HER2+	[157]
Jurkat and supt1/CCR5 cells	CD45+	[185]
Melanoma exosomes	melanocyte antigen A (MelanA)	[180,181]

## Data Availability

Not applicable.
